# Phytochemical Analysis and Allelopathic Potential of an Aggressive Encroacher Shrub, *Euryops floribundus* (*Asteraceae*)

**DOI:** 10.3390/plants14040601

**Published:** 2025-02-17

**Authors:** Masibonge Gxasheka, Zukile Mbita, Kagiso Laka, Mthunzi Mndela, Phesheya Dlamini

**Affiliations:** 1 Department of Plant Production, Soil Science & Agricultural Engineering, School of Agriculture & Environmental Sciences, University of Limpopo, Private Bag X1106, Sovenga, Polokwane 0727, South Africa; phesheya.dlamini@ul.ac.za; 2 Department of Livestock and Pasture, Faculty of Science and Agriculture, University of Fort Hare, Alice 5700, South Africa; mmndela@ufh.ac.za; 3 Department of Biochemistry, Microbiology and Biotechnology, University of Limpopo, Private Bag X1106, Sovenga, Polokwane 0727, South Africa; zukile.mbita@ul.ac.za (Z.M.); kagiso.laka@ul.ac.za (K.L.)

**Keywords:** *Euryops floribundus*, allelopathy, bioactivity, *Lactuca sativa*, phytochemicals

## Abstract

*Euryops floribundus* is an encroaching shrub species that poses a threat to grassland diversity and productivity in the Eastern Cape region of South Africa. This shrub inhibits understory herbaceous plant recruitment and establishment, thereby exposing soils to erosion, owing potentially to toxins it secretes. However, the allelochemicals of *E. floribundus* and their potential effects on the germination and establishment of plants remains poorly understood. We investigated the phytochemical classes of leaves and twigs of *E. floribundus* and evaluated the effects of extracts from these plant parts on seed germination and seedling growth of *Lactuca sativa* through a laboratory experiment. In the laboratory, we analysed phytochemicals in leaf and twig extracts and tested their allelopathic effects on *Lactuca sativa* seed germination and growth using the Petri dish method. In this proof-of-concept study, we identified 12 phytochemical classes of *E. floribundus*. Quantitative analysis showed that the leaves had significantly higher levels of flavonoids, phenolics, and tannins than twigs. As a result, leaf extracts caused 100% inhibition of seed germination, while twig extracts inhibited seed germination by 90% at 50 g L^−1^. Both leaf and twig extracts also significantly reduced radicle and plumule growth, with a stronger effect observed from the leaves than twigs. This study provides new insights into the phytochemical composition and strong allelopathic potential of *E. floribundus*, contributing to a better understanding of the mechanisms driving its encroachment in semi-arid grasslands.

## 1. Introduction

Shrub encroachment is the phenomenon where native shrub and woody plant species increase in density, cover, and biomass beyond their historical or geographical ranges [1,2,3]. This land cover transformation has become a significant concern in arid and semi-arid grasslands and savannas worldwide [4,5,6,7]. Shrub encroachment is linked to reduced forage production, which in turn affects livestock productivity, ultimately impacting the livelihoods of farmers [1,7,8]. Despite the widespread of shrub encroachment, its underlying causes remain poorly understood, though it is largely attributed to changes in fire regimes, overgrazing, erratic rainfall, increases in carbon dioxide and global climate change [8,9,10,11]. Beyond these general factors, there is a lack of studies investigating allelopathy as a contributing factor towards shrub encroachment, despite its significant influence on ecosystem structure and function in other contexts [12,13].

Allelopathy is often referred to as any effect, either direct or indirect, positive or negative, of one species on the development of another via the release of chemical compounds into the environment [12]. Some encroaching plant species produce an array of phytochemical classes, including phenolics, coumarins, tannins, cinnamic acid, quinones, steroids and terpenoids, glycosteroids, and alkaloids [14,15,16]. Many of these compounds are inherently allelopathic and play a crucial role in the self-defence mechanisms of plants [17,18]. Allelopathic compounds are gradually released into the environment through various mechanisms, including volatilization or leaching from aerial parts, exudation from roots, and decomposition of plant residues in the soil [17,19]. Plant species belonging to the *Asteraceae* family have been documented to employ allelopathy as a mechanism for colonising grasslands, fields and roadsides [14,16,17]. The allelopathic phytochemicals of this plant species often exert a deleterious effect on the germination and seedling growth of a target plant [20,21]. For instance, a recent study conducted by Judžentienė et al. [22] reported that the allelopathic effects of *Solidago canadensis* (*Asteraceae*) extracts led to a reduction in the seed germination and growth of *Lactuca sativa* and *Lepidium sativum*. Similarly, Pan et al. [16] observed that *Solidago canadensis* (*Asteraceae*) extracts hindered *Lantana indica* seed germination and seedling growth. Likewise, Kapoor et al. [21] found that *Artemisia absinthium* (*Asteraceae*) extracts efficiently decreased the seed germination and growth of *Parthenium hysterophorus*. Mokou et al. [23] also found that extracts from *Seriphium plumosum* (*Asteraceae*) significantly reduced germination and radicle lengths of *Lactuca sativa*.

In this study, we investigate the phytochemical classes in the leaves and twigs of *Euryops floribundus* N.E. Br and evaluate their effects on the seed germination and seedling growth of *Lactuca sativa*. *E. floribundus* is a South African indigenous plant, which is extensively encroaching in the Eastern Cape Province of South Africa. This shrub species is mostly found in the Chris Hani District Municipality, particularly in areas near Queenstown [24,25,26,27]. *E. floribundus* belongs to the genus *Euryops*, a member of the *Asteraceae* (*Compositae*) family [28]. Worldwide, the family *Asteraceae* comprises over 1000 genera, which together make up a large collection of more than 25,000 species of flowering plants [14,29]. In South Africa, the *Asteraceae* family accounts for 63 endemic species in the flora of the Eastern Cape Province. Earlier research has documented that some species within the *Asteraceae* family, including *E. floribundus*, have an invasive or aggressive encroaching inclination, which negatively impacts the herbaceous plant community [30,31,32]. *E. floribundus* has also been identified as an encroaching shrub species in many areas of the Eastern Cape Province [33,34,35].

The encroachment of *E. floribundus* adversely affects herbaceous vegetation by diminishing the abundance of palatable grasses and forbs [26,36,37]. *E. floribundus* also alters soil properties in encroached grasslands [35,37]. In addition, *E. floribundus* is not palatable to livestock including goats and cattle [36,38]. Furthermore, the shrub species reduces grass cover, which may expose soil to degradation [33]. Taken together, these observations suggest that *E. floribundus* may have a hitherto unexplored allelopathic potential. However, to date, there is no available information on the phytochemical composition of *E. floribundus*, and such empirical data are critical to enhance our understanding of the allelopathic potential of *E. floribundus*. To address this knowledge gap, we conducted a proof-of-concept study using a laboratory experiment aimed at (a) identifying the phytochemical classes present in the plant parts (i.e., leaves and twigs) of *E. floribundus*, and (b) evaluated the effects of extracts from these plant parts on the seed germination and growth of *Lactuca sativa*.

## 2. Materials and Methods

### 2.1. Plant Material Collection in the Field

**Figure 1:**
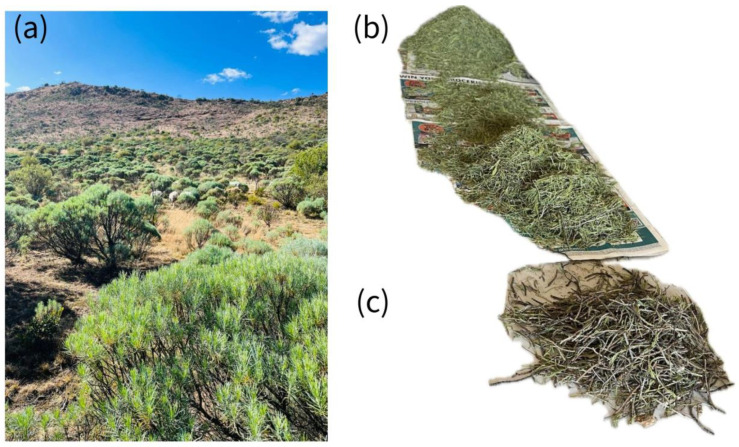
Landscape encroachment by *E. floribundus* (**a**), dry leaves (**b**) and dry twigs (**c**). Photo credits: Masibonge Gxasheka.

In this study, we selected leaves and twigs, as they are the primary plant parts exposed to environmental factors and are likely to release allelopathic compounds either through leaching by rain or through the decomposition of leaves and twig debris after falling to the ground [39]. Thus, leaf and twig samples of *E. floribundus* were randomly collected from mature shrubs, with an average height of 1.3 m and a canopy area of 1.4 m^2^, before flowering in communal grasslands located in Tsengiwe [40], Eastern Cape Province, South Africa (S 31°35′13.1″, E 27°38′07.5″; Figure 1a–c). The collected leaf and twig samples were placed in brown paper bags and transported to the laboratory for further processing.

### 2.2. Handling and Preparation of Plant Parts 

In the laboratory, the collected leaf and twig samples were first unpacked from the paper bags and carefully inspected for any debris or contaminants. Leaf and twig samples were then air-dried at room temperature for two weeks. The air-dried leaves and twigs were then ground to powder using a waring commercial laboratory blender. The prepared leaf and twig samples were then stored in airtight containers and kept refrigerated at 4 °C for less than a week before the experiment.

### 2.3. Phytochemical Analysis

#### 2.3.1. Phytochemical Screening in the Laboratory

The analysis was performed to detect the presence of various phytochemical classes. For this purpose, 50 g of milled leaf material and 50 g of twig material were each macerated in 500 mL of tap water at a ratio of 1:10 (*w*/*v*). The solutions were subjected to a vigorous 24 h shaking at room temperature using a shaking incubator machine (New Brunswick Scientific Co., Inc., Edison, NJ, USA) set at 200 revolutions per minute (rpm). Pre-weighed, marked containers were filled with the filtrate of the extracts. To fully extract the constituents, the procedure was performed repeatedly over a period of three hours and one hour. The aqueous extracts from each mixture were then mixed. The solvent was evaporated under a stream of cold air using a commercial fan. The preliminary phytochemical screening of the leaves and twig extracts was conducted using standard procedures to detect the active compounds [41,42]. The compounds that were screened for included proteins, phenols, tannins, flavonoids, steroids, cardiac glycosides, quinones, anthraquinones, leucoanthocyanins, coumarins, saponins, alkaloids, carbohydrates, cholesterol, lignin and resins. A positive reaction to these tests was defined as the formation of a precipitate or the appearance of any colour change (refer to Table 1 for more information). The tests were performed in triplicate and independently repeated three times.

#### 2.3.2. Quantitative Analysis of Phytochemicals

From the screened phytochemicals, total tannins, total phenolics, and total flavonoids were selected for quantification based on their known bioactive properties and allelopathic effects [43,44].

##### Total Phenolic Content (TPC)

Using the Folin–Ciocalteau method, the total phenolic content (TPC) amount in the acetone-dissolved extract was determined using a previously published protocol [45]. Briefly, a mixture of 100 µL extract and 900 µL distilled water was prepared in a 15 mL centrifuge tube. To this mixture, 100 µL of Folin–Ciocalteu (FC) reagent was added, and the samples were incubated at 25 °C for 1 h and 30 min. Following incubation, 1000 µL of 7% sodium carbonate (Na_2_CO_3_) was added to each sample, and the volume was adjusted to 2500 µL with distilled water. Gallic acid was used as a standard compound and processed in the same manner as the experimental samples. After incubation at 25 °C for 1 h and 30 min, the absorbances were measured at 550 nm using a microplate reader (Promega, Madison, WI, USA). The experiment was conducted in triplicate and independently repeated, three times.

##### Total Tannin Content (TTC)

The Folin–Ciocalteau method was used to determine the tannin content [46]. In short, 100 µL of the acetone-dissolved extract at various concentrations were added to 2500 µL of distilled water in 15 mL centrifuge tubes. Thereafter, 100 µL of 2 M FC reagent and 500 µL of 35% Na_2_CO_3_ solution were added to the mixtures. The final volume was adjusted to 5000 µL with distilled water, and the samples were incubated at 25 °C for 30 min. Standard solutions comparable to the experimental samples were prepared using tannic acid. Absorbances were then measured at 725 nm using a microplate reader (Promega, Madison, WI, USA). The experiment was performed in triplicate and independently repeated, three times.

##### Total Flavonoid Content (TFC) 

The previously reported procedure [47] was applied in order to measure TFC. In summary, incubation was conducted for 30 min at 25 °C for 100 µL of each concentration, including 100 µL of 10% aluminium chloride, 100 µL of 1 M potassium acetate, and 2800 µL of distilled water. Quercetin was used as the standard compound. Absorbance was measured at 415 nm using a microplate reader (Promega, Madison, WI, USA). The experiment was carried out in triplicate and independently repeated, three times.

### 2.4. Allelopathic Evaluation of E. floribundus

#### 2.4.1. Preparation of Aqueous Extracts for Bioassays

Leaf and twig extracts were prepared by adding 25 g and 50 g of air-dried material from each plant part to 1 L of distilled water [31]. These concentrations were chosen to represent the potential allelopathic effects of young and mature shrubs as described by Snyman [31] who studied a similar encroaching shrub species from the *Asteraceae* family. The 25 g concentration represents a low concentration of plant material contribution, associated with young shrubs, while the 50 g concentration reflects a higher contribution by mature shrubs. The extracts were shaken every 6 h and left at room temperature for 24 h. Afterwards, the aqueous extracts were filtered twice using cotton and then twice through Whatman No. 1 filter paper. The resulting extracts were stored in a refrigerator at 4 °C throughout the duration of the experiment. In this study, we used the water extraction approach, since allelopathic interference is known to arise mainly from water-soluble compounds released into the environment [48,49].

#### 2.4.2. Laboratory Bioassays for Allelopathic Evaluation

To explore the allelopathic potential of *E. floribundus*, a bioassay was conducted using lettuce (*Lactuca sativa*) as the model species. This chosen model species is extensively employed in allelopathic studies due to its heightened sensitivity to allelopathic compounds, rapid germination, and uniformity [50,51]. *Lactuca sativa* seeds were purchased commercially from a local market, Makro (Polokwane, South Africa). The seeds were surface-sterilised with 1% NaOCl for 10 min and washed with distilled water. Twenty-five seeds were evenly distributed on Whatman no. 1 filter paper in three 90 mm plastic Petri dishes, resulting in three replicates per treatment. The seeds and filter paper were moistened with 5 mL of either extract solution and distilled water served as the control. The Petri dishes were sealed with paraffin film to prevent water loss and contamination. Subsequently, they were placed in an incubator set at 25 °C under dark conditions for a period of 7 days [52,53]. The number of germinated seeds was counted every day for seven days, and radicle protrusion of 1 mm was used as a criterion for successful germination. After seven days, ten robust seedlings per Petri dish were randomly chosen to measure seedling growth (radicle length, plumule length, and seedling length) using a calibrated ruler. In this study, we investigated seed germination through germination indices (germination rate, germination index, and seed vigour index) as previously performed by Zhao et al. [54], which were calculated as follows:

Germination rate (GR):

(1) $$\mathrm{GR}\left(\%\right)=\left(\frac{\mathrm{Ni}}{N}\right)\times 100$$

where Ni is the number of germinated seeds on the 7th day, while N is the total seed number in the Petri dish.

Germination index (GI):

(2) $$\mathrm{GI}=\sum \left(d/n\right)$$

where d is the number of germinated seeds on a specific day, and n is the time after setting the seeds for germination.

Seed vigour index (SVI):

(3) $$\mathrm{VI}=\mathrm{GR}\times \left(\mathrm{RL}+\mathrm{PL}\right)$$

where GR is germination rate, RL is the radicle length, and PL is the plumule length.

To evaluate the intensity of allelopathic effect, allelopathic response index (RI) was determined using the following formula [55]:

(4) RI=1−(C/T)(T≥C),  RI=T/C−1(T<C)

where T is the mean value of each extract treatment and C is the mean value of the control. RI > 0 denotes growth promotion, RI < 0 indicates inhibition, RI = 0 means no discernible effect, and the magnitude of RI values represents the strength of the allelopathic effect. The synthesis effect (SE) of allelopathy was determined using the following formula [56]:

(5) $$SE=\left({RI}_{GR}+{RI}_{RL}+{RI}_{PL}\right)/3$$

where RI_GR_ is the RI value of the germination rate, RI_RL_ is the RI value of the radicle length and RI_PL_ is the RI value of plumule length.

## 3. Statistical Analysis

The normality of the data was evaluated using the Shapiro–Wilk test, and the homogeneity of variances were assessed by Levene’s test. Owing to the lack of normal distribution, the Kruskal–Wallis test was employed to investigate the influence of *E. floribundus* extract concentrations on seed germination indices (GR, GI and VI), growth characteristics (RL, PL, and SL), and the allelopathy index (RI and SE). The results were deemed significant at *p* ≤ 0.05. Subsequently, a post hoc analysis was conducted using the Dunn’s multiple comparison test. In addition, the mean differences in phytochemical groups (i.e., flavonoids, phenolics, and tannins), germination indices, growth parameters and allelopathy indices (RI and SE) between plant parts were tested using an independent *t*-test, with differences considered significant at *p* < 0.05. Statistical analyses were carried out using R software version 4.3.1 [57]. Figures were generated using GraphPad Prism version 8.0 for Windows (GraphPad Software, Boston, MA, USA).

## 4. Results

### 4.1. Screening and Quantitative Phytochemical Analysis of Leaves and Twigs of E. floribundus

**Figure 2:**
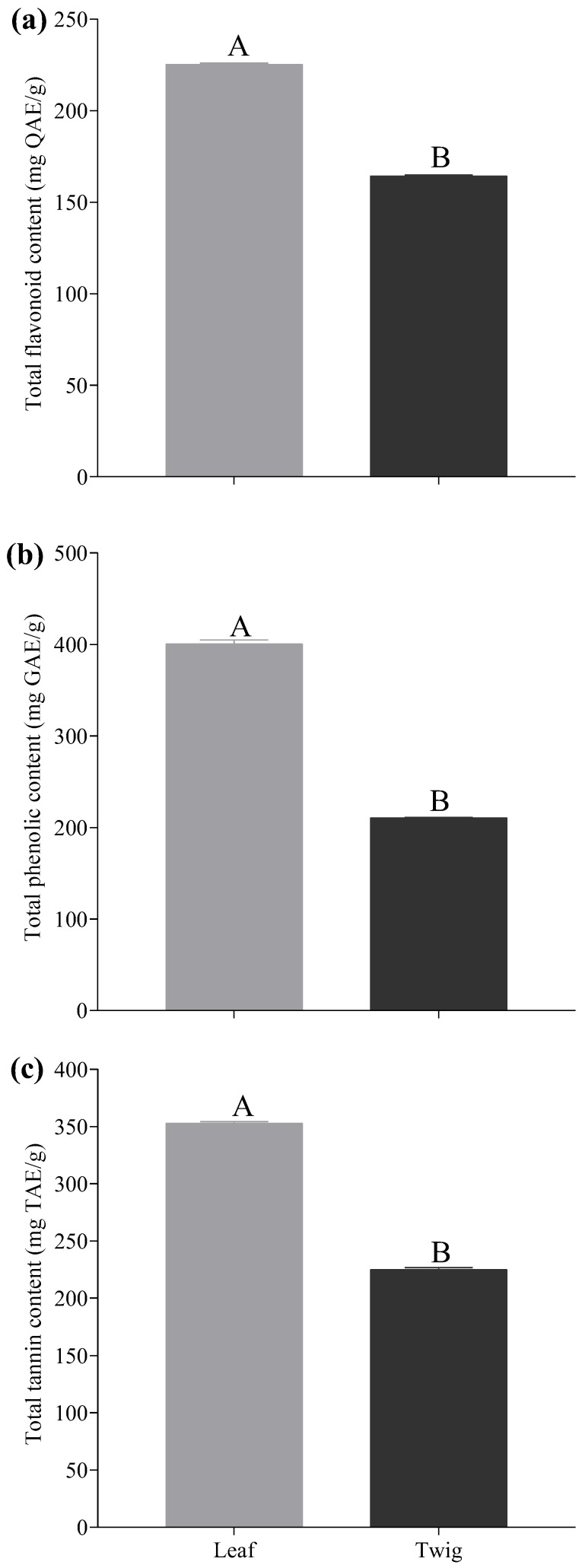
Quantitative analysis of (**a**) total flavonoids, (**b**) total phenolics, and (**c**) total tannins in leaves and twigs of *E. floribundus*. Data are presented as mean ± SD (n = 3). Different capital letters indicate significant differences (*p* < 0.05) between plant parts. For details regarding SD, see Supplementary Table S1. Note: SD = Standard Deviation; n = number of replicates (n = 3).

In the present work, we screened for 16 phytochemical classes on the leaves and twigs of the shrub *E. floribundus*. Only, 12 different phytochemical classes were detected in the leaf and twig extracts of *E. floribundus* (Table 2). In particular, the leaf extract showed a high concentration of tannins, and moderate levels of phenols, flavonoids, steroids, cardiac glycosides, quinones, coumarins, and carbohydrates. Furthermore, the leaf extract had lower amounts of alkaloids, resins, and saponins. In contrast, anthraquinones, leucoanthocyanins, cholesterol, lignin, and resins were absent from the leaf extract. In the twig extract, a high concentration of phenols, leucoanthocyanins, carbohydrates, and coumarins was observed, while flavonoids, tannins, and alkaloids showed moderate levels. Additionally, saponins were present in small amounts in the twig extract. Conversely, no anthraquinones, cholesterol, steroids, cardiac glycosides, quinones, lignin, and resins were present in the twig extract. The quantitative analysis of phytochemical classes showed significant differences in the concentrations of flavonoids, phenolics, and tannins among the leaves and twigs of *E. floribundus* (Figure 2). The leaves contained significantly more flavonoids (225 ± 0.61 mg QAE/g) than the twigs (164 ± 0.44 mg QAE/g) (*p* < 0.05; Figure 2a). Similarly, the leaves showed significantly higher amounts of phenolics (401 ± 3.97 mg GAE/g) and tannins (353 ± 1.57 mg TAE/g) than the twigs, which had 211 ± 0.40 mg GAE/g of phenolics and 225 ± 1.74 mg TAE/g of tannins (*p* < 0.05; Figure 2b,c).

### 4.2. Effects of E. floribundus aqueous Extracts on Seed Germination of Lactuca sativa

**Figure 3:**
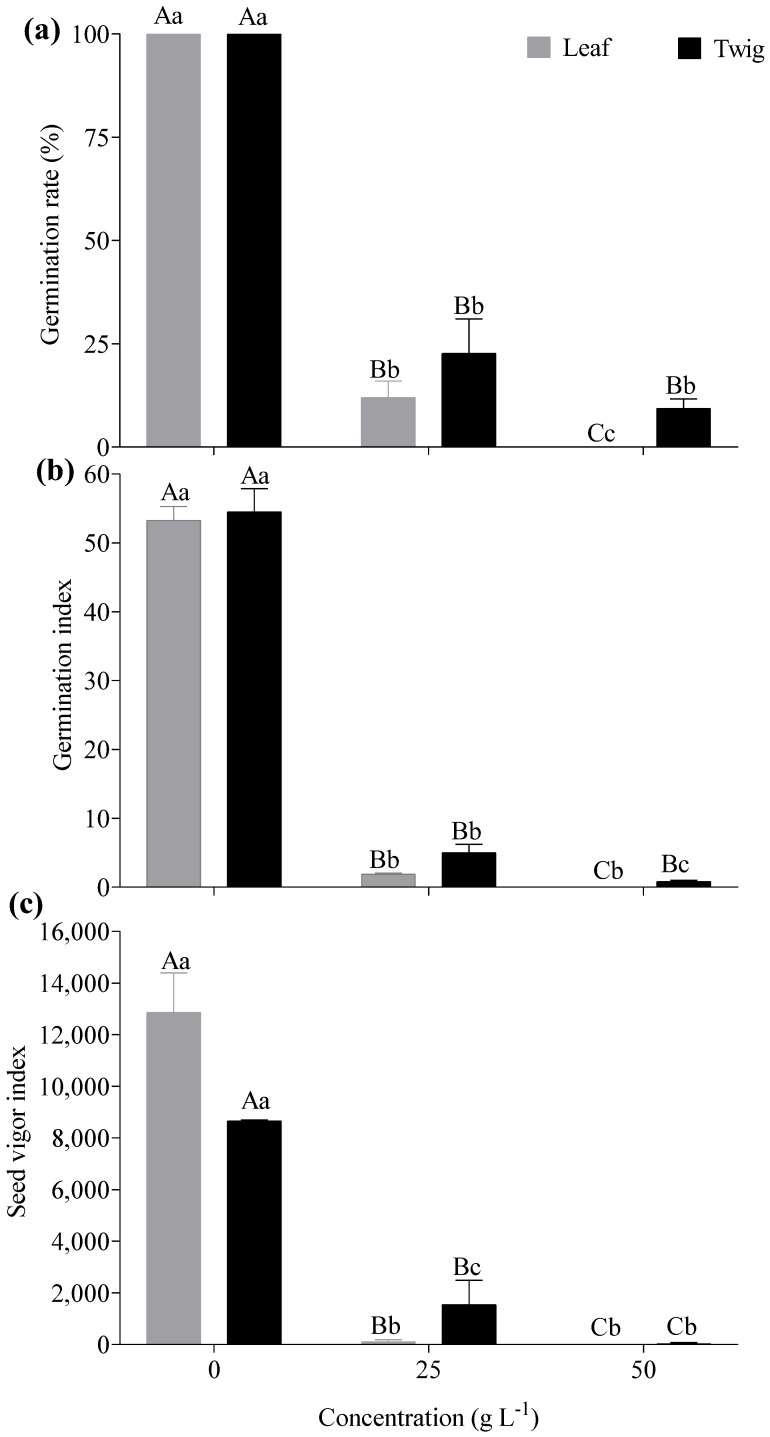
Effects of different aqueous extracts of *E. floribundus* on (**a**) germination rate, (**b**) germination index and (**c**) seed vigour index of *Lactuca sativa*. Data are presented as mean ± SD (n = 3). Different capital letters for the same organ indicate significant differences among different concentrations at the *p* ≤ 0.05 level, while different lowercase letters of the same concentrations indicate significant differences between different plant parts at the *p* ≤ 0.05 level. Note: SD = Standard Deviation; n = number of replicates (n = 3).

The effect of the leaf and twig aqueous extracts of *E. floribundus* on the seed germination of *Lactuca sativa* varied with concentration (*p* < 0.05; Figure 3). Notably, germination indices decreased with an increase in leaf and twig extract concentrations (Figure 3a–c). On average, the leaf extract showed lower germination rates of 12% and 0% at 25 g L^−1^ and 50 g L^−1^ concentrations, respectively, compared to 0 g L^−1^, with a 100% germination rate (Figure 3a). Similarly, twig extracts significantly reduced germination rate by a magnitude of 4- and 10-fold at 25 and 50 g L^−1^ relative to 0 g L^−1^. In leaf and twig extracts, the germination index decreased at 25 g L^−1^ and 50 g L^−1^ concentrations, with leaf extracts showing index values of 1.9 and 0, while twig extract had 5 and 0.8, respectively, compared to 0 g L^−1^ with a germination index of 53.3 and 54.5, respectively (Figure 3b). Similarly, leaf and twig extracts significantly reduced the seed vigour index at concentrations of 25 g L^−1^ and 50 g L^−1^, with index values declining to 104 and 0, respectively, compared to the control concentration of 0 g L^−1^, which had a seed vigour index of 12867 and 11867, respectively (Figure 3c).

### 4.3. Effects of Aqueous Extracts of E. floribundus on Growth Characteristics of Lactuca sativa

**Figure 4:**
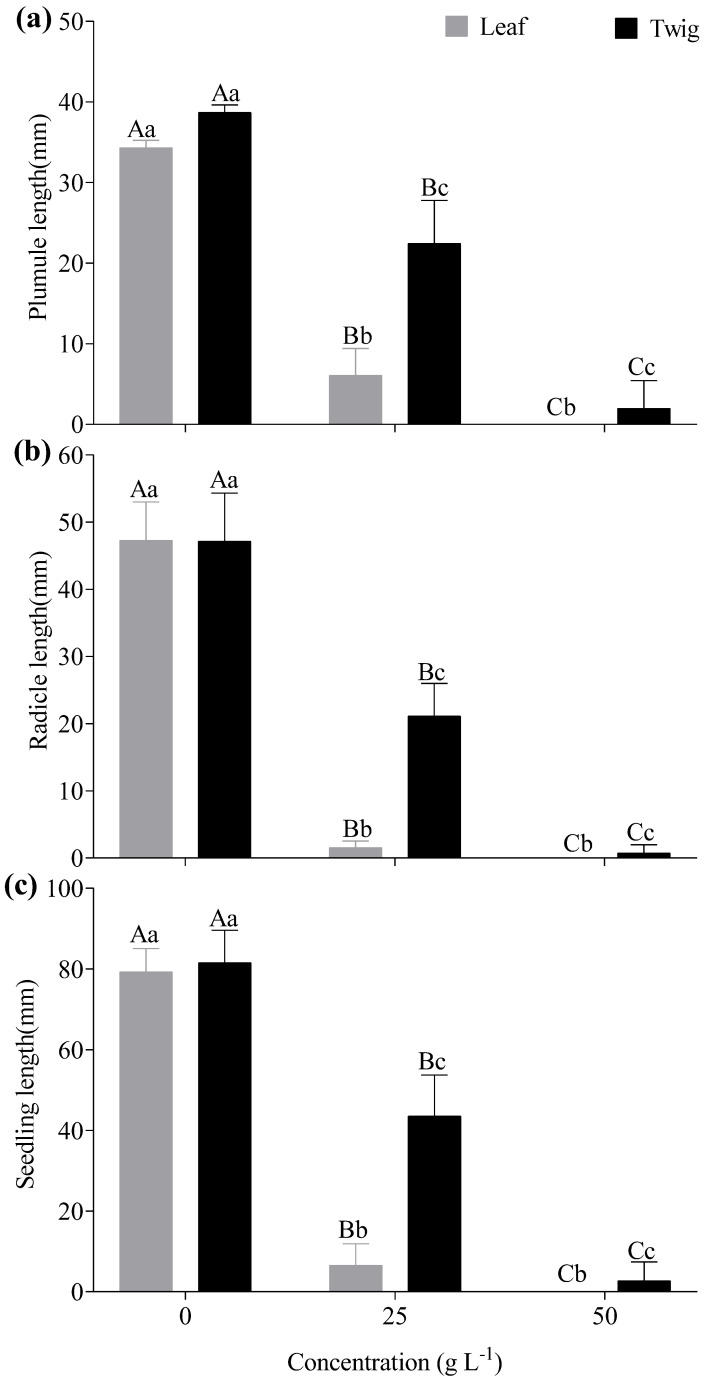
Effects of different aqueous extracts of *E. floribundus* on (**a**) plumule length, (**b**) radicle length, and (**c**) seedling length of *Lactuca sativa*. Data are presented as mean ± SD (n = 3). Different capital letters of the same seedling growth parameter show significant differences among different concentrations at the *p* ≤ 0.05 level, while different lowercase letters of the same concentrations indicate significant differences between different plant parts at the *p* ≤ 0.05 level. Note: SD = Standard Deviation; n = number of replicates (n = 3).

Aqueous extracts of *E. floribundus* significantly affected the growth of *Lactuca sativa* (*p* < 0.05; Figure 4). The plumule length, radicle length, and seedling length decreased with increases in leaf and twig extract concentrations (Figure 4a–c). In particular, leaf extracts drastically decreased the plumule length at 25 g L^−1^ and 50 g L^−1^ concentrations (with an average of 6.08 mm and 0 mm, respectively) compared to the 0 g L^−1^ (34.33 mm; Figure 4a). Likewise, twig extracts decreased the plumule length at the same concentrations (by an average of 22.43 mm and 1.99 mm, respectively) compared to the control concentration, with a plumule length of 38.71 mm (Figure 4a). Similarly, leaf and twig extracts substantially reduced radicle length at 25 g L^−1^ and 50 g L^−1^ concentrations, with an average of 1.55 mm and 0 mm for leaf extract and 21.13 mm and 0.72 mm for twig extracts, respectively, compared to the control concentration of 0 g L^−1^, with a radicle length of 47.28 mm for leaf extracts and 47.17 mm for twig extracts (Figure 4b). At concentrations of 25 g L^−1^ and 50 g L^−1^, seedling length also declined, with means of 6.49 mm and 0 mm for leaf extract, and 43.56 mm and 2.71 mm for twig extracts, compared to the control concentration of 0 g L^−1^, with seedling lengths of 79.32 mm and 81.50 mm, respectively (Figure 4c).

### 4.4. Effect of Aqueous Extracts of E. floribundus on Allelopathic Response Index (RI) and Allelopathy Synthesis Effect (SE)

**Figure 5:**
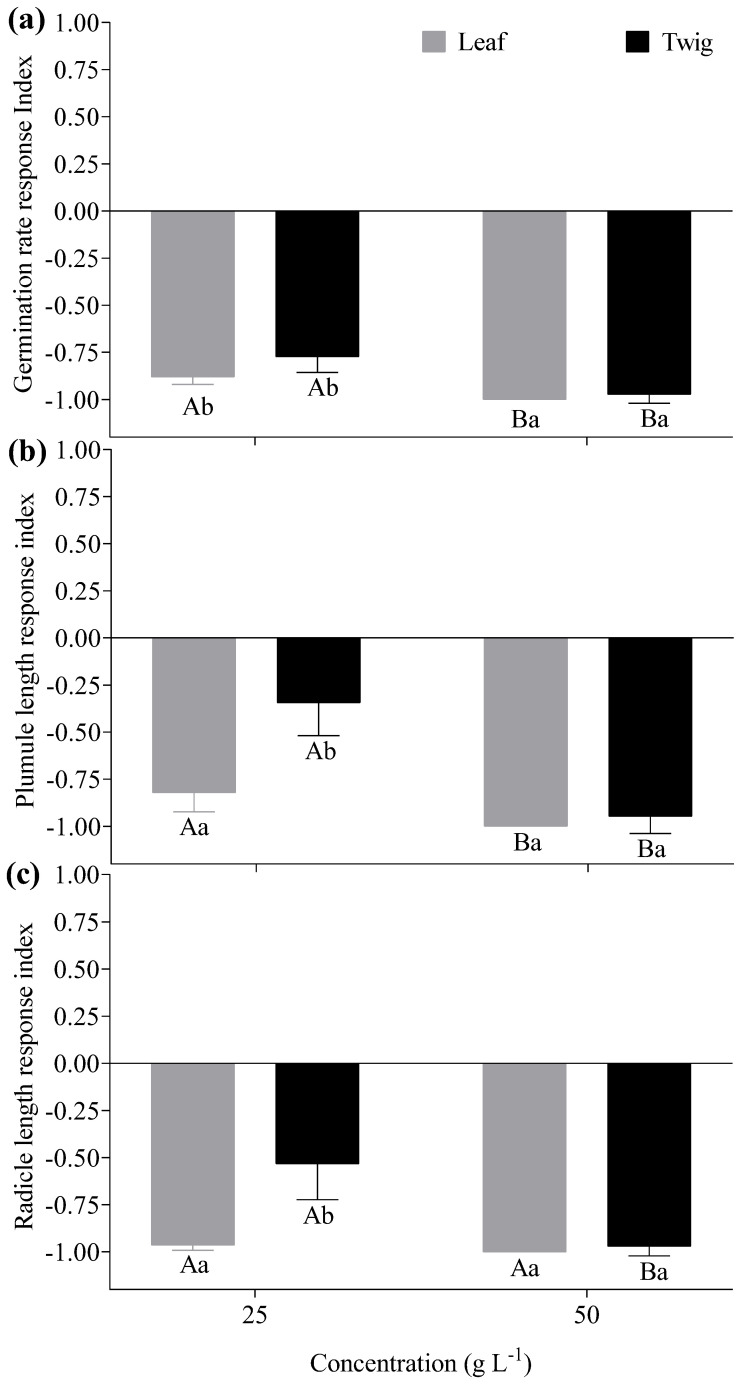
Effects of different aqueous extracts of *E. floribundus* on the allelopathic response index (RI) for (**a**) germination rate, (**b**) plumule length and (**c**) radicle length of *Lactuca sativa*. Data are presented as mean ± SD (n = 3). Different capital letters regarding the same seedling growth parameter show significant differences among different concentrations at the *p* ≤ 0.05 level, while different lowercase letters of the same concentrations indicate significant differences between different plant parts at the *p* ≤ 0.05 level. Note: SD = Standard Deviation; n = number of replicates (n = 3).

**Figure 6:**
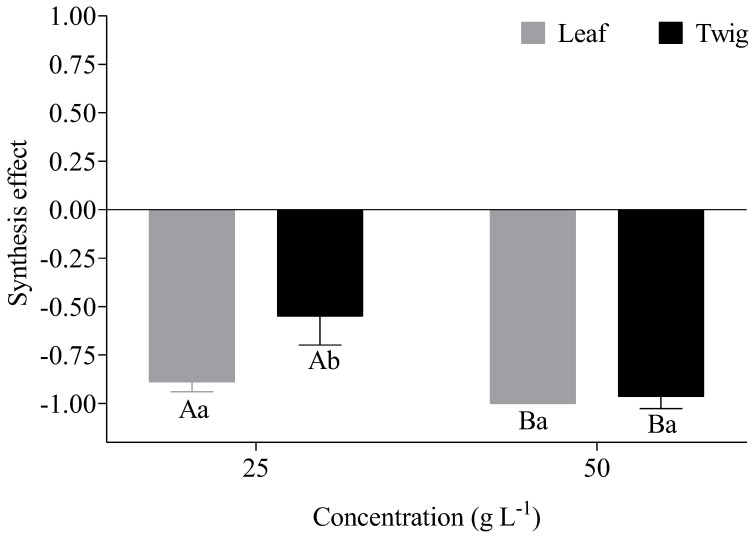
Effects of different aqueous extracts of *E. floribundus* on the synthesis effect (SE) of allelopathy. Data are presented as mean ± SD (n = 3). Different capital letters regarding the same seedling growth parameter indicate significant differences among different concentrations at the *p* ≤ 0.05 level, while different lowercase letters in the same concentrations indicate significant differences between different plant parts at the *p* ≤ 0.05 level. Note: SD = Standard Deviation; n = number of replicates (n = 3).

The allelopathic indices of *Lactuca sativa* were significantly influenced by the aqueous extracts of *E. floribundus* (*p* ≤ 0.05; Figure 5). Noticeably, the allelopathic indices were less than zero, indicating that the extracts of *E. floribundus* suppressed seed germination and seedling growth of *Lactuca sativa*. In the leaf exacts, the RI of germination rate, plumule length, and radicle length significantly decreased as the extract concentrations increased (*p* ≤ 0.05; Figure 5a–c). In particular, the RI of the germination rate was significantly higher at a 50 g L^−1^ (−1.00) concentration than a 25 g L^−1^ (−0.88) concentration for leaf extracts (*p* ≤ 0.05, Figure 5a). Similarly, the twig extract showed a significantly higher germination rate RI at a 50 g L^−1^ (−0.97) concentration compared to a 25 g L^−1^ (−0.77) concentration. Regarding the plumule length, RI was significantly greater at a 50 g L^−1^ (−1.00) concentration compared to a 25 g L^−1^ (−0.82) concentration for the leaf extract, while the RI was also significantly higher at a 50 g L^−1^(−0.95) concentration than a 25 g L^−1^ (−0.32) concentration for the twig extract (*p* ≤ 0.05, Figure 5b). In leaf extracts, the RI for radicle length was not significantly different between the 50 g L^−1^ (−1.00) concentration and 25 g L^−1^ (−0.96) concentration (*p* ≥ 0.05, Figure 5c). On the contrary, the RI for radicle length was significantly (*p* ≤ 0.05) greater at a 50 g L^−1^ (−0.97) concentration compared to the 25 g L^−1^ (−0.53) concentration for twig extract. The allelopathy synthesis effect (SE) was significantly greater (*p* ≤ 0.05) at the 50 g L^−1^ (−1) concentration in comparison with the 25 g L^−1^ (−0.88) concentration for leaf extract (*p* ≤ 0.05, Figure 6). Likewise, the SE of the twig extract was significantly greater at the 50 g L^−1^ (−0.96) concentration compared to the 25 g L^−1^ (−0.55) concentration.

## 5. Discussion

### 5.1. Phytochemicals of Leaf and Twigs of E. floribundus

Phytochemicals, also referred to as secondary metabolites, are naturally occurring bioactive chemical compounds found in plants [58,59,60]. These compounds are extremely diverse chemically and are crucial to the development, physiology, and stress tolerance of plants [58,61]. In the past decades, a broad spectrum of phytochemicals in the *Asteraceae* family have been documented, and their occurrences have been seen to vary depending on the genus, plant species and plant parts [29,60,62]. In this study, we found a range of phytochemical groups in the aqueous extracts of *E. floribundus*, including phenols, tannins, flavonoids, steroids, cardiac glycosides, quinones, leucoanthocyanins, coumarins, saponins, alkaloids, and carbohydrates. In particular, our study found that the leaves of *E. floribundus* contain higher concentrations of total phenolics, total tannins, and flavonoids compared to the twigs. This finding aligns with the results of Wyatt et al. [63] and Ladhari et al. [64], who also observed higher concentrations of phytochemicals in the leaves compared to the twigs in *Kunzea ericoides* and *Ficus carica* species. In contrast, Cheikhyoussef et al. [65] and Kubczak et al. [66] found no significant difference in phytochemical content between leaves and twigs in *Myrothamnus flabellifolius* and *Rosa canina* species.

Additionally, the higher concentrations of phytochemical classes in the leaves of *E. floribundus* are consistent with findings from earlier research on the *Asteraceae* family, which suggests that these plants primarily store bioactive phytotoxins in the epidermal and mesophyll cells of their leaves [67,68,69]. For example, Harouak et al. [70] reported a variety of phytochemical classes such as tannins, terpenoids, reducing compounds, Anthracene derivatives, and alkaloids in leaves of *Artemisia herba alba* Asso. Wahua and Pepple [71] also found that diverse presence of phytochemicals such as saponins, flavonoids, terpernoids, cardiac, cardiac glycosides, alkaloids, aglycone glycosides, steroids, tannins, and phenols in the leaves of *Tridax procumbens*. Moreover, it is crucial to highlight that the accumulation of phytochemical classes is regulated by a range of factors, including the variety, morphology, and genetics of a given plant species, as well as the time of the year [69,72]. Consequently, these factors may contribute to the diverse presence and concentration of phytochemical classes in *Asteraceae* plant species, explaining the observed various phytochemical concentration in *E. floribundus*.

### 5.2. Effects of Aqueous Extracts of E. floribundus on Seed Germination of Lactuca sativa

In this research, we explored the allelopathic potential of the shrub *E. floribundus* on seed germination of *Lactuca sativa*. Our results provide compelling evidence of the allelopathic effects of *E. floribundus* aqueous extract on the seed germination of *Lactuca sativa*, with stronger effects observed at higher concentrations. Notably, leaf extracts from the *E. floribundus* shrub species showed a lethal allelopathic effect with 100% inhibition while twig extracts exhibited a 90% inhibition on seed germination at 50 g L^−1^, which was the highest concentration tested. One possible explanation for the observed deadly inhibition on seed germination of *Lactuca sativa* is the presence of higher concentration of phytochemical classes such as phenols, tannins, and flavonoids on the leaves of *E. floribundus* (Figure 2). These compounds may have a fatal effect on seed germination by disrupting cell membrane function and altering permeability, interacting with hormonal processes, causing changes in enzymatic activities, interfering with cell division and compromising the overall development of the embryo [64,73,74,75]. This lethal reduction in seed germination by aqueous leaf extracts has also been documented in other allelopathic woody plant species (i.e., *Castanea henryi*, *Retama raetam*, *Halocnemum strobilaceum* and *Limoniastrum guyonianum*) [76,77]. In recent research, Bouafiane et al. [77] showed that higher concentrations of aqueous aerial extracts obtained from *Halocnemum strobilaceum* and *Retama raetam* totally suppressed the germination of *Lactuca sativa* seeds. Similarly, Li et al. [78] found that aqueous leaf extracts of *Artemisia Argyi* exerted a deadly inhibition on seed germination of targeted species (*Lactuca sativa* and *Brassica pekinensis*) at higher concentration. Likewise, Ming et al. [76] investigated the aqueous leaf extract of *Castanea henryi* and found that greater concentrations inhibited seed germination in the tested species (*Brassica pekinensis*). Zhang et al. [79] also found that the aqueous leaf extracts of *Koelreuteria bipinnata* var. *integrifoliola* exerted a deadly inhibition on the seed germination of targeted species (*Agrostis tenuis*) at higher concentrations. Ultimately, the phytotoxins produced by *E. floribundus* may inhibit the germination and establishment of understory vegetation. These phytochemicals are likely to create an unfavourable environment for seedling growth, potentially preventing the successful germination of competing plant species. Such inhibitory effects could lead to a decline in plant diversity, favouring the growth of the shrub itself while limiting the regeneration of other herbaceous species. This disruption in plant community dynamics may contribute to shifts in ecosystem structure and function, particularly in grassland areas where *E. floribundus* is prevalent.

### 5.3. Influence of Leaf and Twig Extracts of E. floribundus on Growth Characteristics of Lactuca sativa

In this study, the lengths of both plumules and radicles showed a strong declining trend with increases in aqueous extract concentrations. Specifically, leaf and twig extracts of *E. floribundus* showed more pronounced influences on radicle growth than on plumule growth in *Lactuca sativa*. These findings align with the results of other allelopathic studies, which consistently reported a more pronounced impact of allelopathy on radicle length than on plumule length [80,81,82,83]. For example, Wang et al. [84] observed that Canada goldenrod extracts had a greater effect on the root length than the leaf length in *Lactuca sativa*. Similarly, the study conducted by Yu et al. [85] reported a strong effect of *Solidago canadensis* extracts on the radicle length compared to the plumule length of *Lactuca sativa*. In our study, the observed reduction in radicle and plumule length hinges on our phytochemical analysis, which showed a greater abundance of phenols, tannins and flavonoids in leaf and twig exacts of *E. floribundus* (Table 2). This greater concentration of phytochemical classes may contribute to the suppression of plumule and radicle growth in *Lactuca sativa*. These phytochemical classes govern plant growth and development by affecting phytohormone activity and cell division [86,87]. In fact, these phytochemical classes alter the distribution of hormones such as auxin, gibberellic acid, and ethylene, thereby hindering the growth and development of recipient plants [78,88]. Furthermore, a recent study by de Alcântara et al. [83] showed that the presence of phenolic compounds interfered with the growth of the radicle and caulicle of *Lonchocarpus sericeus*. Consequently, the reduction in radicle and plumule development affects the competition capacity and vegetative establishment of *Lonchocarpus sericeus* seedlings.

### 5.4. Allelopathic Effects of Aqueous Extracts of E. floribundus

The overall allelopathic effect of *E. floribundus* on *Lactuca sativa* was assessed using a response index (RI) analysis, which included germination rate, plumule length, and radicle length. The RI values for the different extract concentrations in different plant parts of *Lactuca sativa* were all negative, illustrating that aqueous extracts of the *E. floribundus* are allelopathic to *Lactuca sativa*. The synthesis effects (SE) of allelopathy were also negative, with their absolute values increasing with the increase in extract concentrations. These findings are consistent with the findings of Li et al. [77], who studied the allelopathic effect of *Artemisia argyi* and found negative RI values for different plant parts for the target species (i.e., *Lactuca sativa* and *Brassica pekinensis*) along with negative SE values for different aqueous extracts of *Artemisia argyi*. Ma et al. [89] also reported negative RI values of different plant parts of *Lactuca sativa*, as well as negative SE values in different plant parts of *Mikania micrantha* with increasing extract concentrations. Furthermore, leaf extracts were more allelopathic than twig extracts at the tested concentrations. In line with our findings, Ming et al. [76] observed that the leaves of *Castanea henryi* demonstrated greater allelopathic effects on *Brassica pekinensis* compared to twigs and shells. In our study, the strong allelopathic effects of leaves can be attributed to the increased presence of phytochemical classes, such as phenols, tannins, flavonoids, steroids, cardiac glycosides, quinones, and coumarins, which are documented as influential allelochemicals [44,69,90].

Although our results provide strong evidence of the allelopathic effects of *E. floribundus*, it is worth noting that lettuce (*Lactuca sativa*) was used as a high-sensitivity model species [49,50] to detect potential allelopathic activity. These results do not necessarily reflect interactions with native or endemic plant species, which may show different tolerance levels. Future research should investigate the responses of ecologically relevant species to gain a more comprehensive understanding of allelopathic effects in the natural environment.

## 6. Conclusions

This study provides the first documented evidence of the allelopathic potential and phytochemical composition of *E. floribundus*. The findings obtained in this study revealed that *E. floribundus* has allelopathic potential on the germination and seedling growth of the model species (*Lactuca sativa*), with an overall inhibitory effect. Specifically, we found that the germination indices and growth characteristics of *Lactuca sativa* decrease as extract concentrations increase. Additionally, leaf extracts demonstrated a more pronounced lethal allelopathic effect compared to twigs at higher concentrations of *E. floribundus* aqueous extracts. Our research identified a diverse range of phytochemical classes in *E. floribundus*, including phenols, tannins, flavonoids, steroids, cardiac glycosides, quinones, coumarins, saponins, and alkaloids. Furthermore, the leaves were found to contain higher amounts of flavonoids, phenolics and tannins compared to the twigs, which may contribute to the observed effects of leaf extracts. Taken together, our findings suggest that allelopathy could be a significant contributor to the encroachment of *E. floribundus* in communal grasslands. We encourage further studies to identify and quantify the specific allelochemical species causing the observed phytotoxicity. Additionally, further studies should evaluate specific, identified allelochemical species in native grass species through pot and field experiments. This knowledge is crucial for improving our understanding of *E. floribundus* allelopathic potential, which will allow for the proper development of a management strategy to restore grassland diversity as well as offers a potential for bioherbicide development and pharmacological research.

## Figures and Tables

**Table 1:** Procedure for preliminary phytochemical screening of aqueous *E. floribundus* extracts.

Phytochemicals	Test	Procedure	Observation
Phenols	Ferric chloride test	2 mL extract + 2 mL of 2% ferric chloride	Blue/black colouration
Tannins	-	2 mL extract + 2 mL H_2_O + 3 drops of 5% FeCl_3_	Green precipitate
Flavonoids	Ferric chloride test	1 mL extract + 1 mL 10% FeCl_3_	Yellow colouration
Saponins	-	-	-
Steroids	-	2 mL extract + 2 mL CHCl_3_ + 2 mL H_2_SO_4_	Reddish brown ring
Alkaloids	Tannic acid test	1 mL extract + 10% tannic acid	A buff colour precipitate
Cardiac glycosides	-	-	-
Proteins	-	1 mL extract + 2 mL H_2_SO_4_	White precipitate
Anthraquinones	-	-	-
Leucoanthocyanins	Isomyl alcohol test	2 mL + 2 mL isomyl alcohol	Organic layer into red
Coumarins	NaOH test	2 mL extract + 3 mL 10% NaOH	Yellow colouration
Quonones	HCL test	1 mL extract + 1 mL HCL	Green colour
Carbohydrates	Test for starch	1 mL extract + 2 mL 5% KOH	A canary colouration
Cholesterol	-	2 mL extract + 2 mL chloroform + 10 drops of acetic anhydride + 3 drops H_2_SO_4_	A rose-red colour
Lignin	Labat test	1 mL extract + 1 mL gallic acid	Olive green colour
Resins	Acetic anhydride test	1 mL extract 1 mL acetic anhydride + 1 mL H_2_SO_4_	Orange to yellow colour

**Table 2:** Preliminary phytochemical analysis of aqueous *E. floribundus* extracts.

Phytochemicals	Leaf Exact	Twig Extract
Phenols	++	+++
Tannins	+++	++
Flavonoids	++	++
Steroids	++	-
Cardiac glycosides	++	-
Quinones	++	-
Anthraquinones	-	-
Leucoanthocyanins	-	+++
Coumarins	++	+++
Saponins	+	+
Alkaloids	+	++
Carbohydrates	++	+++
Proteins	-	-
Cholesterol	-	-
Lignin	-	-
Resins	_+_	-

Note: (+++) Stronger intensity reaction; (++) strong intensity reaction; (+) weak intensity reaction; (-) not detected.

## Data Availability

The data are not publicly available due to confidentiality agreements. However, data inquiries can be directed to the corresponding author.

## Supplementary Materials

The following supporting information can be downloaded at: https://www.mdpi.com/article/10.3390/plants14040601/s1, Table S1: Descriptive statistics (mean ± SD, n = 3) for quantitative analysis of phytochemical classes in the leaves and twigs of *E. floribundus*.
