# Supplementary material for: Phytochemical Analysis and Allelopathic Potential of an Aggressive Encroacher Shrub, Euryops floribundus (Asteraceae)
Source: Plants (Basel). 2025 Feb 17;14(4):601. doi: 10.3390/plants14040601 (PMC11859777; doi:10.3390/plants14040601)
Supplement: Supplementary file 1 [file plants-14-00601-s001.zip › plants-3453241-supplementary.pdf]

Supplementary Material

Table S1. Descriptive statistics (mean ± SD, n = 3) for quantitative analysis of phytochemical classes in the leaves and twigs of *E. floribundus*.

Phytochemical class	Leaf (Mean ± SD)	Twig (Mean ± SD)
Total flavonoids	225±0.61	164±0.44
Total phenolics	401±3.97	211±0.40
Total tannins	353±1.57	225±1.74

Note: SD = Standard Deviation; n = Number of replicates (n = 3).
